# Differences in codon bias cannot explain differences in translational power among microbes

**DOI:** 10.1186/1471-2105-6-3

**Published:** 2005-01-06

**Authors:** Les Dethlefsen, Thomas M Schmidt

**Affiliations:** 1 Department of Microbiology and Molecular Genetics, Michigan State University, East Lansing, Michigan 48824, USA; 2 Department of Microbiology and Immunology, Stanford University, Palo Alto, California 94304, USA

## Abstract

**Background:**

Translational power is the cellular rate of protein synthesis normalized to the biomass invested in translational machinery. Published data suggest a previously unrecognized pattern: translational power is higher among rapidly growing microbes, and lower among slowly growing microbes. One factor known to affect translational power is biased use of synonymous codons. The correlation within an organism between expression level and degree of codon bias among genes of *Escherichia coli *and other bacteria capable of rapid growth is commonly attributed to selection for high translational power. Conversely, the absence of such a correlation in some slowly growing microbes has been interpreted as the absence of selection for translational power. Because codon bias caused by translational selection varies between rapidly growing and slowly growing microbes, we investigated whether observed differences in translational power among microbes could be explained entirely by differences in the degree of codon bias. Although the data are not available to estimate the effect of codon bias in other species, we developed an empirically-based mathematical model to compare the translation rate of *E. coli *to the translation rate of a hypothetical strain which differs from *E. coli *only by lacking codon bias.

**Results:**

Our reanalysis of data from the scientific literature suggests that translational power can differ by a factor of 5 or more between *E. coli *and slowly growing microbial species. Using empirical codon-specific *in vivo *translation rates for 29 codons, and several scenarios for extrapolating from these data to estimates over all codons, we find that codon bias cannot account for more than a doubling of the translation rate in *E. coli*, even with unrealistic simplifying assumptions that exaggerate the effect of codon bias. With more realistic assumptions, our best estimate is that codon bias accelerates translation in *E. coli *by no more than 60% in comparison to microbes with very little codon bias.

**Conclusions:**

While codon bias confers a substantial benefit of faster translation and hence greater translational power, the magnitude of this effect is insufficient to explain observed differences in translational power among bacterial and archaeal species, particularly the differences between slowly growing and rapidly growing species. Hence, large differences in translational power suggest that the translational apparatus itself differs among microbes in ways that influence translational performance.

## Background

Translational power is the rate of protein synthesis of a cell or culture, normalized to the amount of biomass invested in the protein synthesis machinery. We are introducing the term 'translational power' to describe precisely the same concept (and the same quantitative parameter, see Methods) that was originally defined as 'ribosome efficiency' [1-3]. In recent years, this concept has more commonly been called 'translational efficiency' [4,5], particularly in discussions of codon usage bias [6-8]. Although we are reluctant to depart from established terminology, we do so to avoid an inconsistency with the meaning of 'efficiency' as it is used in many other areas of science and in common parlance. In the physical sciences and in many areas of biology, the efficiency of a process refers to a comparison of output to input, in particular to the fluxes of useful energy and/or mass (e.g., the efficiency of a heat engine [9], trophic transfer efficiency [10]). These scientific meanings of 'efficiency' are consistent with the common notion that a process obtaining the desired output with little waste is highly efficient.

According to these conventions, calculations of efficiency make no direct reference to the *rate *at which a process occurs. Physicists and engineers use a distinct term, 'power,' to refer to the rate of energy consumption or the rate at which work is performed [11]. The semantic distinction between power (or rate) and efficiency is important, because in many real and idealized physical systems, the laws of thermodynamic result in an inherent tradeoff between power and efficiency [9]. In biology, several attempts to argue for the universality of power-efficiency tradeoffs [12,13] have justifiably been criticized for the misapplication of thermodynamic arguments [14-16]. Nonetheless, many specific tradeoffs have been demonstrated in a wide range of organisms that can be described as evolutionary choices between power (increased rates of biological processes such as resource acquisition, metabolism or organismal growth) and efficiency (increased biological output measured as probability of survival, production of biomass, number of progeny, etc. per unit resource) [17-24]. Among bacteria, comparisons of coexisting species or strains have also provided evidence for power-efficiency tradeoffs [25-28], as have comparisons of engineered mutant strains [29,30]. However, the absence of apparent tradeoffs in some carefully designed studies of bacteria demonstrates that such tradeoffs are not inevitable [31-33]. Even if power-efficiency tradeoffs occur only in some biological contexts, it is valuable to maintain a semantic distinction between power (implying rapid rate) and efficiency (implying low waste).

However, the terms 'ribosome efficiency' and 'translational efficiency' blur this distinction, because they refer to a rate – the quantitative measure of ribosome efficiency [1] is expressed in units of (time^-1^). We prefer the term 'translational power', which refers to the rate of protein synthesis of a cell or culture, normalized to the mass of the translational apparatus, in a manner that is more consistent with the connotations of 'power' and 'efficiency' derived from other areas of science and from colloquial usage. Translation rate (a synonym of 'protein chain growth rate' [3,34], meaning the rate of amino acid polymerization per active ribosome) is one component of translational power, but translational power reflects other properties of the protein synthesis system as well, most notably the fraction of ribosomes that are active (see Methods, also chapter 6 of reference [34]). Intuitively, translational power measures the capacity of the protein synthesis subsystem to drive replication of the cell, the protein-dominated autocatalytic system to which it belongs.

The concept and a quantitative metric of translational power were first introduced to facilitate comparisons of translational performance between different growth rates within a single bacterial strain [1]. The initial belief that translational power is nearly constant in a strain across a wide range of growth rates, based both on empirical data and theoretical arguments [2,34], has gradually given way to the current understanding that translational power increases with growth rate, at least in *E. coli *[3,4,35,36]. The question of whether translational power varies between microbial species has been investigated only rarely, in four studies that each compared a single slowly-growing microbial species to *E. coli *[37-40]. In each case, translational power was found to be higher in *E. coli *than in the slowly growing comparison strain. Although each of these studies discusses this unexpected result, only one of them references the same result from another study. In previous work, the consistent association of low translational power with slowly growing microbes appears to have escaped notice; however, our reanalysis of the data from these four studies as well as additional published data (presented in Results) suggests that the association is robust.

One factor capable of affecting translational power is the biased usage of synonymous alternative codons. In the standard translational code, 18 of the 20 amino acids are encoded by more than a single codon, but in many microorganisms, synonymous codons are not used with equal frequency. The pattern first found in *E. coli *and *Bacillus subtilis *turns out to be common: the majority of genes within an organism show a preference for the same subset of codons, but the degree of bias towards the preferred subset is correlated with the expression level of the gene [41,42]. For some time, the consensus has been that such a pattern reflects selection for translational power [7,8]. Codon bias increases translational power because preferred codons tend to be translated more rapidly than synonymous alternatives [43-45]. This effect can be attributed to the high abundance of tRNAs cognate to the preferred codons, to a canonical base pair interaction at the codon wobble position between preferred codons and their cognate tRNAs, or to both these factors [7,8]. Codon bias resulting from selection for translational power (or for any other translation-dependent benefit) is correlated with gene expression level because the benefit accrues during each instance of translation, so the selective pressure for preferred codons is stronger in more highly expressed genes [7,8].

In contrast to the codon bias caused by translational selection, codon bias that is consistent in both magnitude and direction in genes that vary widely in expression level is explained most easily by mutational bias acting on DNA [8,46]. While the effects of both translational selection and mutational bias are evident in some microbial genomes with moderately biased G+C content [47,48], organisms with strong mutational bias (very high or low G+C content) have been reported to show very little [49] or no [50-52] evidence of translational selection. Theoretical calculations indicate that if the strength of mutational bias exceeds a certain critical threshold, any pre-existing codon preferences that conflict with the mutational bias will be reversed [53]. In this case, codon use is almost entirely determined by the mutational bias, which influences genes equally regardless of expression level. Note that while the degree of codon bias and the gene expression level would not be correlated among genes from such a genome, this does not necessarily imply that deviations from the average (biased) codon usage would be selectively neutral, nor that the fitness effects of any such deviations would be independent of gene expression level.

The absence of a correlation between codon usage and gene expression level has also been reported in some organisms with moderate G+C content, in particular the spirochete *Treponema pallidum *[54] and the proteobacteria *Helicobacter pylori *[55]. The lack of evidence for translational selection in these organisms requires an explanation, since they lack a strong mutational bias that could obscure such evidence. It has been suggested that rapid exponential growth confers little or no fitness benefit in these strains [8,55], consistent with their slow growth rate and other characteristics of their ecological niche. If so, these organisms would not experience selection for translational power.

If variation in the strength of selection for translational power leads to differences in the degree of codon bias between microbes (superimposed on any differences in codon bias that can be attributed to variation in mutational bias), we wondered whether differences in codon bias could in turn explain the observed differences in translational power between microbes. An estimate of the effect of biased codon use on the overall rate of translation would depend on knowledge of absolute or relative translation rates *in vivo *for each codon. Unfortunately, these data are incomplete even for *E. coli*, and are not available for other microbes. Therefore, we approach the issue by framing the following question: How much faster is the translation rate of *E. coli *than the expected translation rate of a hypothetical organism that has the same proteome composition and the same investment in translational machinery as *E. coli*, but which lacks codon bias? Here we report results from a simple mathematical model developed to address this question.

For convenience, we will refer to the hypothetical *E. coli*-like organism with uniform use of synonymous alternative codons as 'Uni'. By 'same proteome composition', we mean that over a cell generation, each amino acid is incorporated into protein the same number of times in Uni and in *E. coli*, although for the 18 amino acids specified by multiple codons, the individual codons will differ in frequency. By 'same investment in translational machinery', we mean that the total biomass of the translational apparatus is the same in Uni and in *E. coli*, although ideally the allocation of that biomass among various components of the apparatus in Uni would be optimized for unbiased codon usage. However, in order to apply empirical codon-specific translation rate data, we will impose a more stringent requirement on Uni, that the abundance of each individual component of the translational apparatus will be unchanged in comparison to *E. coli*. Due to this restriction, and due to the incomplete data for codon-specific translation rates, we make no claim to be able to answer our question precisely. However, our approximations are adequate to conclude that differences in codon bias alone are unlikely to account for differences in translational power of the magnitude inferred from macromolecular analysis of slowly growing and rapidly growing microbes.

## Results

### Comparisons of translational power among microbes

We know of 4 studies that have made explicit comparisons of translational power between different microbial species; in each case, the comparison was made between *E. coli *and a single slowly growing strain [37-40]. One of these studies relied on original measurements of *E. coli *[38]; the remaining studies made comparisons using the *E. coli *data of Bremer and Dennis [3]. Although growth rates and translation rates vary with temperature [56], at least 2 of the 4 studies [39,40] compared data from strains grown at different temperatures without compensating for temperature effects. One of 2 studies that made comparisons based on the number of ribosomes per cell volume appears to have assumed that *E. coli *cell volume is constant over a range of growth rates [39], which is unlikely. We have reanalyzed the data from these studies (as described in Methods) to provide consistent comparisons of translational power between *E. coli *and other strains. In addition, we applied the same comparative methodology to every microbial species for which we could find the requisite data in the literature. The list of species that could be included is surprisingly short; most studies reporting both the protein and RNA content of microbes growing at known rates have involved *E. coli *or closely related enteric bacteria. Table 1 summarizes the comparisons of translational power between *E. coli *and all other species.

The comparisons of translational power in Table 1 are based on the fastest growth rate for which data are available for each of the comparison organisms, because at submaximal growth rates, there may be a reduction in the average translation rate [4,57], in the active fraction of ribosomes [35,36], or both. Either of these phenomena would reduce translational power. However, the comparisons to *E. coli *are not always based the fastest *E. coli *growth rate, but rather on the growth rate at which *E. coli *makes a comparable investment in the translational apparatus as the comparison organism. A comparison at similar investment levels reflects the expectation that the selective pressure to maximize translational power increases with the biomass invested in the apparatus [4,58]. If the comparisons had always been made to the fastest *E. coli *growth rate (i.e., where *E. coli *translational power is highest), the disparity in translational power would be greater for most of the comparisons shown.

Even with the conservative comparisons displayed in Table 1, the published data suggest that translational power varies considerably between strains, particularly for comparisons between microbes adapted to different ranges of growth rates. While translational power is higher in *E. coli *and other rapidly growing organisms, it is lower in slowly growing organisms, ranging from less than 17% to 42% of the value for *E. coli*. Hence, if differences in the degree of codon bias are to explain these differences in translational power, we would expect codon bias to be capable of accelerating the rate of translation by 2.5-fold to 6-fold. In summarizing the comparisons of Table 1 as a contrast between slowly growing and rapidly growing microbes, we are not relying on the actual growth rates shown in the third column, especially since chemostat growth rates are necessarily constrained below the maximal growth rate for a strain. Instead, we have relied both on well-recognized growth characteristics for some species (e.g., *Sphingopyxis alaskensis *and *Rickettsia prowazekii *are slow growers, *Salmonella enterica *and *Enterobacter aerogenes *are rapid growers), and on the number of copies of the ribosomal RNA (*rrn*) operon per genome. High *rrn *copy number is an adaptation permitting rapid growth [59,60], while low *rrn *copy number is characteristic of microbes adapted for slow growth [39,61].

### Estimates of the translation rate benefit of codon bias

We define the translation rate benefit of codon bias in *E. coli *as *s*_*bias*_, the fractional increase in the time required to replicate the *E. coli *proteome if the actual codon bias of *E. coli *were to be replaced with uniform use of synonymous codons (Equation (10) in Methods). Our estimates of *s*_*bias *_depend on the relative translation rates of individual codons *in vivo*, and on the frequency with which each codon is used in synthesizing the proteome. The sources we have used for these data, and the details of several adjustments made to the source data, are described in the Methods section. All data used in our estimates of *s*_*bias *_are presented in Table 2. Because the codon-specific translation rate data are incomplete even for *E. coli*, we have explored 4 different scenarios (described in Methods) for extrapolating from the empirical rate data to obtain an estimate of *s*_*bias *_over all codons. Scenarios 1–4 are increasingly complex, and represent deliberate attempts to assign translation rates to the unmeasured codons in a way that increases *s*_*bias *_while remaining consistent with patterns found in the empirical data. Furthermore, in Scenario 5, we apply a theoretical approach [62] for predicting optimal codon-specific translation rates that does not rely on empirical translation rate measurements at all, but only on codon frequency and tRNA abundance data.

**Figure 1:**
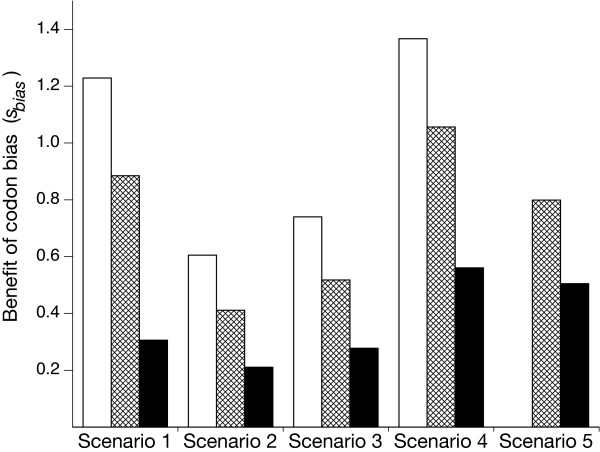
**Translation rate benefit of codon bias in *E. coli ***The estimated translation rate benefit of codon bias in *E. coli*, according to 5 different scenarios (described in Methods) for extrapolating from incomplete empirical data to obtain an estimate over all codons. White bars: duration of translocation assumed to be negligible in comparison to the duration of ternary complex selection. Cross-hatched bars: duration of translocation assumed to be invariant and short in comparison to the duration of ternary complex selection. Both white and cross-hatched bars: benefit of codon bias in *E. coli *estimated relative to uniform codon use. Black bars: duration of translocation assumed to be invariant and short in comparison to the duration of ternary complex selection, benefit of codon bias in *E. coli *estimated relative to a biologically realistic degree of low codon bias (see text).

Estimates of *s*_*bias *_for all scenarios are presented in Figure 1. The empirical translation rate data used in Scenarios 1–4 reflect ternary complex selection at the ribosomal A-site, but not translocation of the newly-formed peptidyl-tRNA from the A-site to the P-site [45]. Thus, for these scenarios we show two estimates of *s*_*bias *_that are based on different assumptions regarding the relative duration of translocation and ternary complex selection. The white bars of Figure 1 are based on the assumption that the duration of translocation is negligible for all codons in comparison to the duration of ternary complex selection. The cross-hatched bars of Figure 1 are based on the assumption that translocation requires a finite amount of time that is constant for all codons, but short in comparison to the time required for ternary complex selection [63]. In Scenario 5 the duration of translocation is not treated explicitly, but the theoretical rate predictions refer to the entire cycle of translational elongation. Hence, we have grouped the estimate from Scenario 5 with other estimates that account for the duration of translocation. Our estimates of the benefit of codon bias in *E. coli *relative to the complete absence of codon bias range from 0.6 – 1.4 if translocation time is neglected, or from 0.4 – 1.1 with the more realistic assumption that translocation requires a short amount of time.

We have also estimated the benefit of codon bias in *E. coli *relative to the limited degree of codon bias that might be found in an actual low-bias organism, rather than making a comparison to the biologically unrealistic standard of strictly uniform synonymous codon use. We took *T. pallidum *as our example of a microbe with limited codon bias, since it is a slowly growing bacterium with little mutational bias (52.7% G+C) that has also been reported to lack translational selection [54]. The *T. pallidum *genome has the second-most uniform codon use over all predicted genes (assessed as Wright's effective number of codons [64]) among 108 bacterial and archaeal species for which complete genome sequences were available in June, 2003 (data not shown). Our method for generating a set of low bias codon frequencies from *T. pallidum *genome codon frequencies is described in Methods. Estimates of the translation rate benefit of codon bias for *E. coli *relative to low bias codon frequencies are shown by the black bars of Figure 1, again assuming a short, invariant duration of translocation. The estimated benefits range from 0.2 – 0.6; as expected, these estimates are smaller than estimates derived from a comparison to strictly uniform codon usage. Because the theoretical estimates of Scenario 5 fall in the middle of the corresponding ranges of empirical estimates from Scenarios 1–4, we are confident that our results are not merely an artifact of unrecognized errors in the empirical rate measurements.

### The benefit of codon bias calculated for individual amino acids

**Figure 2:**
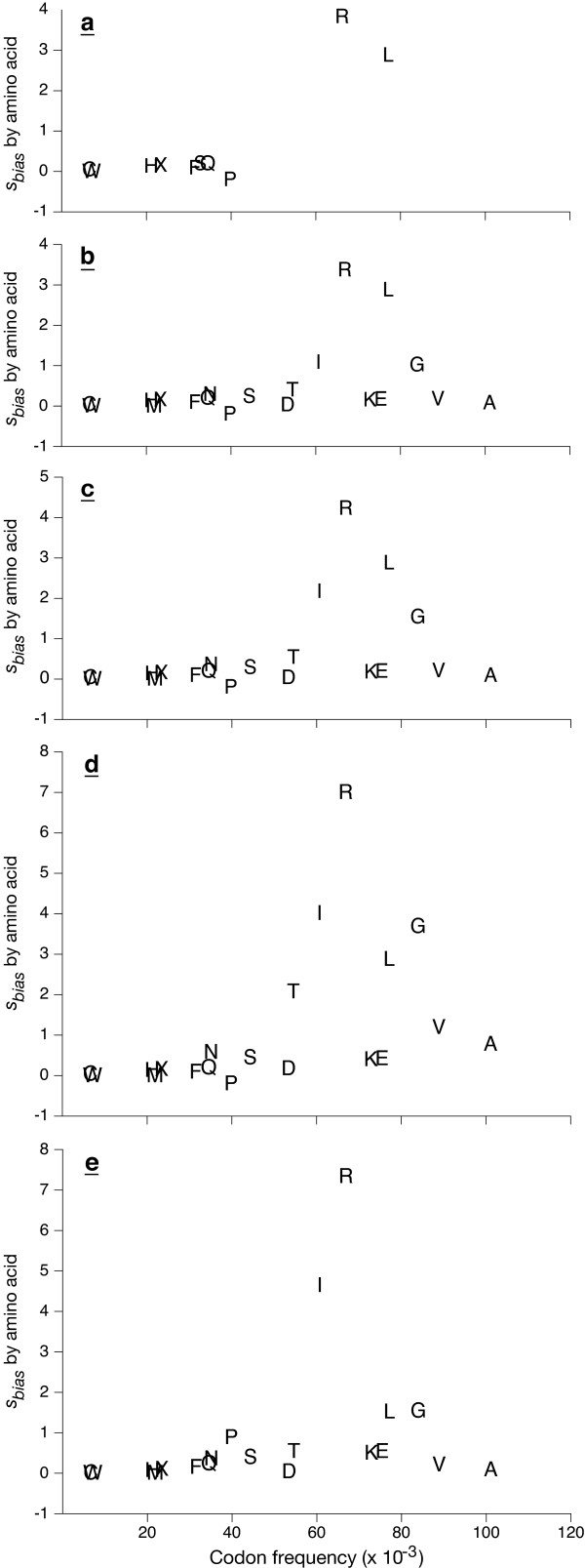
**Translation rate benefit of codon bias by amino acid **The translation rate benefit of codon bias for each amino acid in *E. coli *is plotted versus the frequency of the amino acid in the *E. coli *proteome. Each amino acid is represented by its one-letter abbreviation. Panels a – e represent Scenarios 1 – 5, respectively (described in Methods). For all panels, the duration of translocation is assumed to be negligible and the benefit is estimated in comparison to uniform codon use (corresponding to the white bars of Figure 1). Only a few amino acids encoded by one or more rare codons contribute disproportionately to the total translation rate benefit of codon bias in *E. coli*.

Our definition of *s*_*bias *_can be applied over any subset of codons, in particular, it can be applied to the codons of each amino acid separately. While all amino acids with multiple codons except proline contribute positively to *s*_*bias *_in all scenarios, the magnitude of that contribution is highly variable between amino acids (Figure 2). Codon bias accelerates the translation of most amino acids only slightly in *E. coli*, because most non-preferred codons are not particularly rare in the *E. coli *proteome, compared to the preferred synonym. For example, among the 9 amino acids encoded by 2 codons, on average the preferred codon is 2.9-fold more abundant than the non-preferred codon. Of these amino acids, asparagine shows the greatest difference between preferred and non-preferred codon frequencies, with GAC being 5.2-fold more abundant than GAU. Even if the disparity in codon-specific translation rates is unrealistically large, the ratio of the frequencies of preferred to non-preferred codons in *E. coli *constrains the maximum possible value of *s*_*bias*_. For asparagine, even if the preferred codon were translated instantaneously (i.e., infinitely faster than the non-preferred codon), the difference between using the non-preferred codon at 16% of asparagine residues in *E. coli *instead of at 50% of asparagine residues in Uni corresponds to only about a 3-fold acceleration of translation (*s*_*bias *_≈ 2) for this amino acid. With more realistic disparities between the translation rates of preferred and non-preferred codons, the largest estimate of *s*_*bias *_for asparagine in any of our scenarios is less than 0.2. In other words, we estimate that codon bias in *E. coli *leads to no more than a 20% decrease in the time required to translate all asparagine codons in the proteome (Figure 2).

The amino acids with the largest values of *s*_*bias *_are leucine, isoleucine, and arginine (Figure 2). Although these amino acids are not rare, they possess between them the six rarest codons in *E. coli*, each encoding less than 0.1% of the proteome. (An average codon encodes 1.6% of the proteome.) The frequencies of the most and the least abundant synonyms for leucine, isoleucine and arginine differ by 74-fold, 83-fold, and 1460-fold, respectively. (The higher ratio for arginine reflects the extreme rarity of AGG, which is 17-fold less abundant than the second rarest *E. coli *codon, AUA encoding isoleucine.) Since the translation rates measured or assumed for the 6 rarest codons are quite slow, their increased abundance in Uni accounts for the much of the additional time required for replicating the Uni proteome. If these six codons remained as rare in Uni as they are in *E. coli*, while all other synonymous codons were used without bias in Uni, the translation rate benefit estimated under Scenario 4 (the scenario producing the largest estimate of *s*_*bias*_) would be reduced by almost half (data not shown). The influence of these 6 codons is such that the estimate of *s*_*bias *_is quite sensitive to the translation rates assigned to them, in contrast to the relative insensitivity of *s*_*bias *_to the exact translation rates assigned to most codons.

## Discussion

We want to know whether reduced codon bias could account for the lower translational power measured in at least some slowly growing bacteria, in comparison to *E. coli*. We approach this issue by its converse, calculating how much faster the proteome is replicated in *E. coli *than it would be in the complete absence of codon bias. If we take our estimates at face value, we would conclude that even during rapid growth when the proteome is most biased and translation is fastest, *s*_*bias *_is unlikely to be much larger than 1 (cross-hatched bars of Figure 1), which corresponds to a 2-fold increase in the average translation rate. An effect of this magnitude approaches the smaller disparities in the comparisons of translational power between *E. coli *and slowly growing strains shown in Table 1, but could not explain the roughly 5-fold difference in translational power between *E. coli *and *S. alaskensis*, *R. prowazekii*, *Halobacterium cutirubrum*, or sulfate-reducing strain PT2. However, there are two reasons to think that the benefit of codon bias for *E. coli*, in comparison to most actual slow-growing organisms, is even less than this estimate.

The first reason is that we have prevented our hypothetical Uni from adapting to the codon frequencies we have assigned to it, by keeping the abundance of each component of the translational apparatus fixed. The data do not suggest that maximizing translational power has been the *only *selective pressure influencing codon use in *E. coli *[45,65]. If it had been, the codon with the highest rate constant for ternary complex selection among synonymous alternatives would always be the preferred codon, since it would permit faster translation with a lower biomass investment in cognate tRNA. Of 10 amino acids with multiple codons for which codon-specific translation rate measurements exist [44,45], leucine, serine and proline are not consistent with this prediction. On the other hand, it seems clear that selection for rapid translation has exerted some, and perhaps the major influence on the coevolution of codon frequencies and tRNA abundance in *E. coli*. The codon with the highest rate constant *is *the preferred codon for 7 of the 10 amino acids for which data are available. Other considerations (possibly including error avoidance [66], interactions between adjacent tRNA anticodons [67], or factors unrelated to translation [68]) may have been more influential than the inherent characteristics of the codon-anticodon interactions for determining the preferred codons encoding leucine, serine and proline. However, the importance of rapid translation remains evident in that *E. coli *still translates the preferred codons quickly for 2 of these 3 amino acids, albeit with a larger investment in tRNA than would be necessary if the interaction between the preferred codon and its cognate tRNA occurred more readily.

At a larger scale, the correlation across all codons between frequency and cognate tRNA abundance [69,70] is best explained as a response to selection for rapid translation, as is the pattern of increased bias towards rapidly translated codons with increased levels of gene expression [45]. Without asserting that the distribution of tRNA abundance in *E. coli *necessarily produces the fastest possible translation rate for the *E. coli *codon frequency distribution, it is clear that selection for translational power has been a significant factor in the *co*evolution of codon frequencies and cognate tRNA abundances in *E. coli*. Thus, it is very unlikely that we have attained the maximum possible translation rate for Uni by matching the *E. coli *distribution of tRNA abundance values (in the form of a particular distribution of codon-specific translation rates) to the very different codon frequency distribution of Uni. For this reason, our estimates confound the translation rate benefit of codon bias in *E. coli *with the penalty of a suboptimal allocation of translational resources in Uni.

The second reason that our approach overstates the relative benefit of codon bias for *E. coli *in comparison to actual slow-growing organisms is that actual microbes are not completely devoid of codon bias. Assessing *s*_*bias *_in *E. coli *in comparison to a biologically plausible standard for low codon bias, instead of in comparison to the implausible standard of no codon bias whatsoever, reduces the estimated benefit in *E. coli *by about half (black bars of Figure 1). Only a slight bias in codon use is sufficient to obtain a substantial benefit of faster translation because only a few codons in *E. coli *are translated much more slowly than the median rate (Table 2). Moderate avoidance of only these few codons can provide a considerable acceleration of the average translation rate without generating a dramatic bias in overall codon use.

Our estimate of a biologically plausible standard for low bias codon frequencies is deliberately conservative, underestimating the degree of bias expected in most slowly growing microbes, for two reasons. First, our low bias codon frequencies are based on the *genome *codon frequencies of *T. pallidum*, as if all predicted genes in the genome were expressed equally. Correspondence analysis performed at the level of individual genes failed to uncover evidence that codon use varies with expression level in *T. pallidum *[54]. If this were true, the proteome codon frequencies would indeed be similar to genome codon frequencies, regardless of variability in gene expression levels. However, a more sensitive analysis using codon frequencies summed over a set of putative high expression genes indicates that codon use in such genes is more biased than codon use in the genome as a whole. This conclusion is based on a comparison of Wright's effective number of codons [64] calculated for codon frequencies summed over all predicted genes annotated as ribosomal proteins or translation elongation factors (Nc = 52.7) or calculated for codon frequencies summed over all predicted genes in the genome (Nc = 55.2) [71]. The failure to observe this low level of codon bias in the previous analysis based on individual gene sequences [54] can probably be attributed to high gene-to-gene variability in codon frequency estimates based on the small samples of codons represented by individual genes. Thus, even for *T. pallidum*, the proteome codon frequencies appropriate for estimating the benefit of codon bias will be more biased than the genome-derived low bias codon frequencies shown in Table 2.

The second reason our low bias codon frequencies underestimate the degree of codon bias in most slowly growing microbes is that *T. pallidum *is essentially free of the influence of mutational bias, with a genome G+C content of 52.7%. In contrast, many slow-growing microbes have more extensive codon bias that can be attributed mostly or entirely to the biased nucleotide composition of the genome (e.g., *R. prowazekii *[52], *H. pylori *[55], *Borrelia burgdorferi *[54], *Buchnera aphidicola *[72], *Mycoplasma genitalium *[73], and *Chlamydia *species [74]). If codon bias derived from mutational bias, like codon bias derived from translational selection, permits more rapid translation for the same investment in translational machinery, the use of low bias codon frequencies derived from *T. pallidum *will underestimate the translation rate of many slow growing strains. We believe that codon bias derived from mutational bias does, indeed, have the potential to accelerate translation.

The translation rate benefit of codon bias depends on matching preferred codons with cognate tRNAs that are abundant and/or that form 3 canonical base pairs [7,8]. Even when codon use is determined by mutational bias in the DNA replication and repair systems [46], not by selection acting simultaneously on codons and their cognate tRNAs via translation-associated effects, selection for translational power can influence the relative abundance and anticodon sequence of tRNA species. Relatively few mutations are sufficient to influence the identity and abundance of tRNA molecules in an organism, in comparison to the number of mutations required to influence proteome codon frequencies. (Consider that 45 mutations could allow a single mutation in the anticodon wobble position or in the regulatory region of many or even all tRNA genes, depending on the organism, while 45 mutations could alter the identity of less than 0.5% of the >9,000 codons in genes encoding ribosomal proteins and translational elongation factors.) Hence, the mutation-selection balance argument invoked to explain diminished codon bias in genes expressed at low levels in many strains [8,75] also suggests that the distribution of tRNAs can be influenced by translational selection that may be too weak to create a dramatic effect on codon usage. In fact, if codon use is biased in the same direction in all genes (as expected if the source of codon bias is mutational bias), instead of being biased only in highly expressed genes, it would increase the selective pressure for adaptation of the tRNA pool. Hence, it would be very surprising if the anticodons and the relative abundances of tRNA molecules in organisms with high or low G+C content did *not *reflect their biased use of codons.

This prediction is confirmed by the only two studies we have found of tRNA abundance in microbes with extreme G+C content, involving *Mycoplasma capricolum *(25% G+C) [76] and *Micrococcus luteus *(74% G+C) [77]. *M. capricolum*, but not *M. luteus*, can be considered a constitutively slow-growing strain. As expected, cognate tRNA abundance in both organisms is correlated with codon frequency, both across all codons and within synonymous codon families [76,77]. For *M. capricolum*, this is accomplished largely without the tRNA gene dosage effects that are important for *E. coli *[70] and *B. subtilis *[78], since 28 of the 29 *M. capricolum *tRNA genes are present in only a single copy [76]. These examples indicate that selection for translational power is operative even for organisms in which the codon bias is determined by mutational bias instead of translational selection, and even for slowly growing organisms. Because codon bias from any source can be exploited to obtain higher translational power, the estimates of *s*_*bias *_for *E. coli *compared to codon frequencies derived from *T. pallidum *will overstate the benefit that exists for *E. coli *relative to most other slowly growing microbes that have greater mutational bias.

In summary, we believe the translation rate benefit of codon bias in *E. coli *is likely to be less than 0.6 (see black bars of Figure 1) relative to an actual slow-growing organism that shows limited codon bias, such as *T. pallidum*, and substantially less than 0.6 relative to a slow-growing organism with more extensive codon bias. We do not mean to suggest that the advantage of translating as much as 60% faster than a competitor is unimportant. Clearly, the benefit of codon bias for *E. coli *must be substantial, considering that it arises from the aggregate effect of many thousands of preferred codons that are stably maintained in the *E. coli *genome, despite the randomizing influence of mutation acting at each individual codon. On the other hand, the influence of codon bias on the average translation rate is far smaller than the differences in translational power observed between microbes adapted to different ranges of growth rates. For differences in codon bias to explain the difference in translational power between *E. coli *and *S. alaskensis*, *s*_*bias *_would have to be about 5; to explain the difference between *E. coli *and *R. prowazekii*, *s*_*bias *_would have to be about 3.

Is it possible that the comparisons of translational power presented in Table 1 are flawed? The colorimetric assays used for RNA and protein measurement in these studies are indeed dependent on procedural details, such that comparisons between laboratories and between studies are less reliable than comparisons within a study. Nonetheless, variation between species in the estimates of translational power presented in Table 1 do not appear to result simply from large random errors around a common mean. Estimates of translational power for slowly growing species with few *rrn *operons cluster around low values; the reverse is true for species capable of rapid growth with higher numbers of *rrn *operons. In addition, our own measurements of 10 bacterial species (including *E. coli*, *S. alaskensis *and 8 recent soil bacterial isolates) reproduce the same pattern; we have found differences in translational power that are comparable in magnitude to those shown in Table 1[79]. Hence, we believe the comparisons in Table 1 are an adequate representation of the differences in translational power between rapidly growing and slowly growing microbes.

## Conclusions

Because codon bias influences translational power, and because the degree of codon bias due to translational selection may differ systematically between rapidly growing and slowly growing strains, we investigated the parsimonious hypothesis that observed differences in translational power between microbial species could be explained by differences in the degree of codon bias. However, based on the analysis reported here, such an explanation is not plausible. Instead, differences in translational power between rapidly growing and slowly growing species suggest that the translational apparatus itself has different performance characteristics in rapidly growing and slowly growing microbes.

## Methods

### Translational power, translation rate and the active fraction of ribosomes

Conceptually, we define translational power as the rate of protein synthesis in a cell or culture, normalized to the biomass invested in the protein synthesis system. We intend the term to be synonymous with 'translational efficiency' [4,5,8]; our rationale for departing from established terminology is provided in the Introduction. The protein synthesis system is comprised of ribosomes, elongation factors, tRNAs, tRNA synthetases, mRNAs, and numerous other components. Measuring the mass of the entire system is not trivial, because it includes a variable fraction of the cell's protein. However, since the protein synthesis system includes essentially all the cell's RNA, we follow Kjeldgaard and Kurland [1] in using RNA mass (*R*) as an index of the biomass invested in the entire system. For a culture in balanced, exponential growth, the instantaneous rate of increase of any culture component is d*X*/dt = *μX*, where *μ *is the specific growth rate and *X *is the mass of the component present in the culture at that moment. Hence, *μP *is the rate of protein synthesis in a culture containing mass *P *of protein. Thus, our quantitative measure of translational power is:


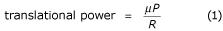


This quantitative measure of translational power will be consistent with the conceptual definition as long as RNA is a nearly constant fraction of the mass of the entire protein synthesis system.

Translational power reflects both the average translation rate and the fraction of active ribosomes in a cell or culture, which we demonstrate as follows, using the approach of chapter 6 of reference [34]. 'Translation rate' refers to the rate of amino acid polymerization of an active ribosome. The average translation rate of a cell or culture is the rate of amino acid polymerization in the entire culture divided by the total number of active ribosomes:


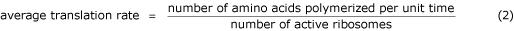


We know that the mass rate of protein synthesis in a culture in balanced growth is *μP*. Units of protein mass can be converted to a number of amino acids by dividing the protein mass by the average mass of an amino acid:

number of amino acids polymerized per unit time = *μP*/(average mass of amino acid)     (3)

The number of ribosomes in a culture containing a mass *R *of RNA can be found by multiplying *R *by the fraction of RNA that is ribosomal, and then dividing by the mass of RNA in a ribosome. However, only a fraction of these ribosomes are active at any given time. Thus:


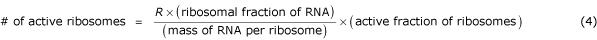


Substituting Equations (3) and (4) into Equation (2) yields:


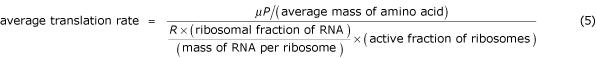


After rearranging terms in Equation (5), we have:


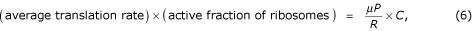


where


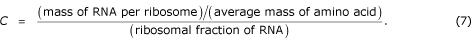


The quantity *μP/R *in Equation (6) is the quantitative measure of translational power from Equation (1) [1,3]. From Equation (6), it is clear that translational power reflects both the average translation rate and the active fraction of ribosomes in a cell or culture.

What of the term we have labeled *C*, implying a constant? The two quantities in the numerator, the mass of RNA in a ribosome and the average mass of an amino acid, are indeed constant or nearly constant, both within a strain at different growth rates, and across strains. However, despite the constant ribosomal fraction of RNA reported in reference [3], other data indicate that the rRNA fraction decreases from about 85% to about 75% as growth rate declines in *E. coli *from 1.7 hr^-1 ^to 0.28 hr^-1 ^[70], a result which is expected on theoretical grounds [4,65]. This variation is not dramatic; it would reduce translational power by only 12%, if the average translation rate and active fraction of ribosomes were unchanged. Data are also available from 2 of the 4 studies that have compared translational power between *E. coli *and a slowly growing strain. The rRNA fraction is reported as 84% for *H. cutirubrum *at specific growth rates of both 0.10 hr^-1 ^and 0.05 hr^-1^, after the authors made the deliberately generous assumption that messenger RNA comprises 5% of the total RNA [37]. The rRNA fraction is about 85% for *R. prowazekii *at a specific growth rate of ~0.07 hr^-1^, after a correction is made for 2–3% messenger RNA [38]. These data suggest that variation between microbial species in the ribosomal fraction of RNA is limited, even when comparing species that grow at very different rates.

### Comparisons of translational power based on published data

Table 1 summarizes comparisons of translational power between *E. coli *and all other bacterial and archaeal species for which we could find both the protein content and the RNA content of cultures growing at known rates. Throughout this work, *E. coli *is represented by the Bremer and Dennis data [3], which are typical of the data reported for *E. coli *in many other studies. Similarly, comparisons between *E. coli *and 2 closely related species of enteric bacteria, *S. enterica *and *E. aerogenes*, are made using only a single representative study for the latter strains, chosen from among several published reports. For the remaining species, only a single published study was available for comparison, except for one species represented by two studies, both of which are included. For strains not grown at 37°C, we assume that the growth rate, but not the macromolecular content, would be altered by growth in the same medium at a different temperature [80]. The growth rates reported for these strains were adjusted to the growth rates expected at 37°C using the linear range of the relationship reported in reference [56]. (Although this temperature-growth rate relationship was generated with *E. coli*, the comparison is mathematically identical whether the temperature correction is applied to *E. coli *or to the comparison strain.)

The comparisons in Table 1 use the fastest growth rate for which data are available for the comparison organisms, and use data for *E. coli *growing at a rate such that it matches the comparison organism for investment in the translational apparatus. (For two of the comparison strains, translational power differed considerably between the fastest growth rates obtained in different culture conditions; both values are reported.) One of three measures was used to gauge the level of investment in the translational apparatus, depending on the quantity measured in the original study. The possible measures were the number of ribosomes per cell volume, the ratio of protein to ribosomal RNA, or the ratio of protein to total RNA. Values of these quantities for *E. coli *were interpolated between adjacent data points to estimate the growth rate at which *E. coli *made the same investment in the translational apparatus as the comparison organism. The translational power of the comparison organism at the fastest available growth rate was then expressed as a percentage of the translational power of *E. coli *at the 'same investment' growth rate. A comparison at similar investment levels reflects the expectation that the selective pressure to maximize translational power increases with the biomass invested in the apparatus [4,58]. If the comparisons had always been made to the fastest *E. coli *growth rate (i.e., where *E. coli *translational power is highest), the disparities in translational power would be greater for most of the comparisons shown.

### Calculation of the translation rate benefit of codon bias

Consider a cell in which a total of *C*_*i *_codons of type *i *are translated during a single cell generation, so that the sum over all sense codons *C *= Σ*C*_*i *_is the total number of codons translated during a cell generation. (Hereafter we refer to the translational output over a cell generation as the proteome.) If we define *c*_*i *_= *C*_*i*_/*C *as the proportion of all codons of type *i *in the proteome and *r*_*i *_as the average translation rate of codons of type *i*, the total time required for replication of the proteome (i.e., the proteome generation time) will be


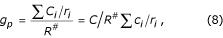


where *R*^# ^is the average number of ribosomes active in translation over the cell cycle and the sum is over all sense codons. Codon bias in favor of rapidly translated codons will reduce *g*_*p *_in comparison to uniform codon use. If a mutation changes the fitness of an organism from *w *to *w*', the benefit of the mutation is typically described as *s*, where *w*'/*w *= 1 + *s*. By analogy, and considering *g*_*p *_to be inversely related to fitness, we can express the translation rate benefit of codon bias as


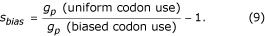


The protein content (and thus *C*) is the same in Uni as in *E. coli *by hypothesis. With the restrictive condition that the abundance of each individual component of the translational apparatus is unchanged in Uni, ribosome content (*R*^#^) will be the same also. Hence, the *C*/*R*^# ^term of *g*_*p *_in Equation (8) cancels from both the numerator and denominator of Equation (9) for *s*_*bias*_, leading to


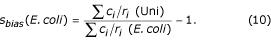


Since amino acid frequencies are identical in *E. coli *and Uni, the disparities in translation rates between synonymous codons largely determine the magnitude of the translation rate benefit of codon bias.

We will use the same codon-specific translation rates (the *r*_*i*_'s) for both Uni and *E. coli*, again invoking the restrictive stipulation that the abundance of each individual tRNA species is unchanged. If rate constants for the interaction of each codon with each of its cognate tRNA species were known, we could calculate the optimal tRNA abundance distribution for the codon frequencies of Uni, and infer the resulting codon-specific translation rates [62,65]. However, *in vivo *codon-specific translation rate data are available only as codon averages, including translation from all tRNA species cognate to each codon. Hence, rate constants specific to each codon-cognate tRNA pair cannot be calculated from the available data for the codons translated by multiple tRNA species, and thus we cannot calculate an optimal tRNA abundance distribution for Uni. Instead, we have constrained Uni to maintain the same tRNA distribution and codon-specific translation rates as *E. coli*. Insofar as the *E. coli *rates reflect an allocation of tRNA abundance that would be sub-optimal for Uni (as we argue in the Discussion section), our approach will tend to overestimate of the benefit of codon bias in *E. coli*, a conservative error for our purposes.

### Data sources

All data used in our estimates of *s*_*bias *_are reported in Table 2.

For the codon frequencies used in synthesizing the proteome of *E. coli*, we rely on the data of Dong *et al*. at a specific growth rate of 1.73 hr^-1 ^[70], compiled from public gene sequence databases and protein abundance data derived from 2D gel electrophoresis studies [81,82]. The absolute codon frequencies shown in Table 2 have been recalculated from [70] with initiation and stop (including selenocysteine) codons removed. As expected, the translation rate benefit of codon bias was found to increase monotonically with growth rate, when calculated by any of the scenarios described below, using the proteome codon frequencies and tRNA abundance data from the range of growth rates reported in reference [70] (data not shown). This increase in *s*_*bias *_reflects simply the increasing bias in both proteome codon usage and relative tRNA abundance with increasing growth rate. Since we are interested in the maximum effect of codon bias, we report results from only the highest growth rate for which data are available.

To investigate the importance of low levels of codon bias, we applied Equation (10) either with Uni having strictly uniform use of synonymous codons, or with Uni assigned a set of low bias codon frequencies (Table 2). The low bias frequencies were generated from relative codon frequencies over all predicted genes in the complete genome sequence of *T. pallidum *[71]. By relative codon frequencies, we mean the absolute frequency of a codon divided by absolute frequency of the amino acid it encodes. The set of *T. pallidum *relative codon frequencies for a particular amino acid were multiplied by the absolute frequency of that amino acid in the *E. coli *proteome; the resulting set of absolute codon frequency values were assigned to the codons of that amino acid in the low bias set so as to retain the same rank order of codon frequency among synonyms as exists in the *E. coli *proteome. For example, the absolute frequency of isoleucine and the identity of the 1st, 2nd and 3rd most common isoleucine codons are the same in the low bias set as in the *E. coli *proteome. However, the relative frequencies of the 1st, 2nd and 3rd most common isoleucine codons in the low bias set are the same as the relative frequencies of the 1st, 2nd and 3rd most common isoleucine codons in the *T. pallidum *genome.

To represent codon-specific translation rates, we use the relative rate data (the quantity R_tRNA_/R_shift_) of Curran and Yarus [45] for the 29 sense codons beginning with U or C (YNN codons, Y = pyrimidine). Although incomplete, this is by far the largest data set available for *in vivo *translational kinetics. The original publication transposed values reported for two arginine codons, CGC and CGA [83]; we have corrected this error. We also revised the rate measured for CGA downward, to account for interference from the bulky wobble position inosine-adenine base pair in the P site that results from translation of a CGA codon. Such interference is strongly suggested to slow selection of a ternary complex at the codon *subsequent *to CGA [83]; such an effect would not have been measured with the experimental system of reference [45], but is appropriate to include as a codon-specific effect of CGA on translation rate. In the absence of more precise data, we reduced the translation rate measured for CGA by a factor of 3, the factor by which CGA reduces read-through of a following stop codon by a suppressor tRNA in comparison to CGC [83]. This adjustment to the CGA rate brings these results into rough agreement with those of Sorensen and Pedersen [84], who used an experimental approach that would have detected a consistent effect of CGA on the translation rate of the subsequent codon, attributing it to slow translation of CGA itself. The relative rates of reference [45], modified as described above, are listed in Table 2.

The relative rates reported by Curran and Yarus [45] do not reflect the entire translational cycle, but rather the time required for selecting a cognate ternary complex at an empty, codon-programmed ribosomal A site, which is believed to occupy the majority of the elongational cycle [63]. Although peptide bond formation may be very rapid, the time required for the EF-G-catalyzed translocation of the ribosome to the subsequent codon (and the associated movement of P- and A-site tRNAs) may not be much shorter than the time needed for EF-Tu-catalyzed ternary complex selection [63]. Hence, in addition to calculations made using rates of ternary complex selection to represent an entire cycle of translational elongation (assuming, in effect, that the duration of translocation is negligible), we also made calculations after modifying the reported rates by adding an invariant 'translocation time' to the variable 'ternary complex selection time' for all codons. The duration of translocation per codon was set at 40% of the average time required to select a ternary complex containing tRNA^phe ^at a UUU codon, consistent with the only quantitative measure of translocation rate that has been made in conditions approximating those *in vivo *[63]. Results from both sets of calculations (white and cross-hatched bars of Figure 1) are presented for each scenario (described below) that is based on these ternary complex selection rates. For convenience, elsewhere in this report we refer to the relative rates of reference [45] as translation rates, rather than using the more accurate but cumbersome expression 'ternary complex selection rates'.

To calculate the total abundance of cognate tRNA for each codon, we assign cognate specificity largely according to Björk [85], and use the tRNA abundance data from Dong *et al*. [70]. We differ from Björk only in assuming that the leucine and glycine tRNAs with uridine in the anticodon wobble position (for which nucleotide modifications have not been characterized) will read codons ending in U, A and G, instead of A and G only. This would be the case if the wobble position U is modified to cmO^5^U, as is done for each of the other 6 amino acids encoded by a full box of the translational code (i.e., amino acids for which the four XXN codons are synonyms). Following Björk, we assume that 40% of the tRNAs for glutamate, glutamine and lysine with uridine in the anticodon wobble position are modified to mnm^5^Se^2^U and thus read codons ending in A or G; the balance of these tRNA species are assumed to have mnm^5^S^2^U in the wobble position and read A-ending codons only [85]. The abundance of two pairs of isoaccepting tRNA species (Gln1 + Gln2 and Ile1 + Ile2) were reported as summed values by Dong *et al*. [70], since these individual species were not separated under the experimental conditions applied. We have resolved the summed values to the abundance of individual species using the ratios of the individual abundance values reported by Ikemura [69]. We show cognate tRNA abundance data in Table 2 as a percentage of total tRNA, omitting initiator and selenocysteine tRNAs; the sum of all values is greater than 100%, reflecting the partially overlapping specificity of many tRNA species.

### Scenarios for extrapolating from incomplete empirical translation rate data

We address the incompleteness of codon-specific translation rate data in several ways. In Scenario 1, we assume that the effects of biased use of YNN codons on translation rate can be used to represent the effects of bias over all codons, without assigning particular translation rates to the unmeasured codons. However, since the YNN codons are almost half of all sense codons but only account for about a third of all expression (Table 2), they must be less highly expressed, on average, than the RNN codons (R = purine). Consequently, selection for translational power may have been weaker among YNN codons than RNN codons. Scenarios 2–4 address this potential deficiency by applying various strategies of assigning translation rates to the unmeasured codons that are consistent with observed patterns, but that could allow the effect of codon bias on translation rate to be greater among RNN codon than YNN codons. Scenario 5 abandons empirical codon-specific translation rate measurements completely, assigning translation rates to all codons on the basis of the proteome codon frequency and cognate tRNA abundance of *E. coli*, assuming optimality (i.e., maximal translation rate) according to theory developed by Solomovici *et al*. [62].

#### Scenario 1

The 29 YNN codons encode 10 amino acids, 9 of which have multiple codons. For 7 of these 9 amino acids, the most common synonym is the codon with the fastest translation rate. One of the remaining amino acids is serine, for which the two fastest-translated codons are the two most abundant, although in reverse order, with relatively small differences between the two in both rate and abundance. Only proline appears to be anomalous; the 2 most abundant codons encode over 90% of all proline residues in the proteome [70], but support ternary complex selection about 3.5-fold more slowly than the 2 least abundant codons [45]. It has been suggested [45] that this anomaly could be adaptive; if proline, because of its unique structure, is found preferentially between protein domains [86] where slow translation may be important to permit cotranslational folding [87,88]. If proline is the only amino acid for which such contrarian selection pressure is more important than selection for translational power, including proline codons in a sample intended to represent all codons will lead to an underestimate of *s*_*bias*_. Hence, in Scenario 1 we apply Equation (10) over YNN codons, with the calculated translation time for non-proline YNN codons weighted by a factor of 3.2, which scales the expression level of these codons to the expression level of all non-proline codons. In other words, we assume the effects of codon bias on translation rate among the 25 non-proline YNN sense codons are representative of the effects of codon bias among all 57 non-proline sense codons, whereas the translation rates measured for proline codons are applied only to themselves.

#### Scenario 2

Curran and Yarus noted that among highly expressed genes, there is a significant tendency for rapidly-translated codons to be used frequently, although the relationship appears to be nonlinear [45]. We observe the same pattern comparing their relative rate data to the proteome codon frequency data of Dong *et al*. [70] at the highest growth rate. For non-proline YNN codons, the best fit (R^2 ^= 0.56) of a quadratic relationship passing through the origin between the codon frequency and translation rate data of Table 2 is *c*_*i *_= 0.205 *r*_*i *_- 0.522 *r*_*i*_^2^. We use this equation to predict translation rates from codon frequency for all RNN codons, as shown in Table 2. Since our objective is to obtain a reasonable estimate the codon-specific translation rate for codons which have not been measured, not to defend a particular model of the relationship between codon frequency and translation rate, we make no attempt to justify a quadratic fit in comparison to other possible functional relationships. The predicted rates for RNN codons and the measured rates for YNN codons (Table 2) are used with Equation (10) to estimate the translation rate benefit of codon bias under Scenario 2.

#### Scenario 3

The preceding scenario applied to the YNN codons tends to predict translation rates among synonymous alternatives that are not as disparate as those actually observed. Furthermore, the fit of a functional relationship between codon frequency and translation rate among YNN codons is better when only preferred codons are considered, instead of all codons. Hence, we fit a quadratic relationship passing through the origin to data from 10 preferred non-proline YNN codons, obtaining *c*_*i *_= 0.352 *r*_*i *_- 1.611 *r*_*i*_^2 ^(R^2 ^= 0.81). Among the 10 preferred codons, we include UGG, the sole tryptophan codon, and UUG, the preferred leucine codon within the UUR split box although not the preferred leucine codon overall. We then apply this equation to predict translation rates from codon frequencies for 12 preferred RNN codons, including AUG, the sole methionine codon, and AGG and AGC, the preferred arginine and serine codons within their respective split boxes, although not the preferred codons overall. For non-preferred RNN codons, translation rate is predicted by multiplying the predicted rate for the preferred synonym (within the full or split box) by the ratio of the square roots of the codon frequencies for the non-preferred and preferred codons:


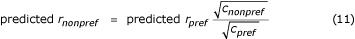


This relationship was chosen both because a dependence on the square root of codon frequency has been suggested repeatedly in theoretical investigations of optimal translation rates [62,65,89,90], and because for all non-preferred RNN codons, this relationship leads to a greater disparity of predicted translation rates compared to the preferred synonym than the regression of Scenario 2. (It also predicts a greater translation rate disparity than is observed for the majority of non-preferred YNN codons.) When both the quadratic regression for preferred codons and Equation (11) for non-preferred codons are applied to predict the translation rate of non-proline YNN codons, the correlation of predicted with measured translation rates is comparable to that attained with Scenario 2 (R^2 ^= 0.57). The predicted rates for RNN codons and the measured rates for YNN codons (Table 2) are used with Equation (10) to estimate the translation rate benefit of codon bias under Scenario 3.

#### Scenario 4

This scenario is generated in three steps, with the goal of generating an estimate of the translation rate benefit of codon bias that is consistent with the most extreme empirical observations. First, three rare RNN codons (AGG and AGA for arginine and AUA for isoleucine, all with *c*_*i *_< 0.1%) are assigned the slowest relative translation rate observed among YNN codons (*r*_*i *_= 0.6 for the rare leucine codon CUA). Second, the translation rates for preferred RNN codons within full or split boxes (except AGG) are estimated according to the regression equation described for Scenario 3. Finally, the translation rates for non-preferred codons (except AGA and AUA) are predicted from the preferred synonym using the ratios of the most disparate translation rates observed empirically among synonymous alternatives, treating split boxes and full boxes of the translational code separately. The most extreme ratio observed among translation rates in a split box is 3.375, for glutamate codons in the study of Sorensen and Pedersen [84]. The most extreme ratios observed for translation rates of codons in a full box is 1:1.3:1.6:24 for the CUN leucine codons in the study of Curran and Yarus [45]. (Exploring other rate values 1 ≤ *x *≤ *y *≤ 24 in ratios of the form 1:*x*:*y*:24 failed to find any that greatly increased the estimated benefit beyond that using the leucine ratios, data not shown.) Although this scenario is based on extreme observations, applying these 3 rules to the non-proline YNN codons leads to a correlation of predicted and measured translation rates (R^2 ^= 0.67) somewhat better than that obtained under Scenario 2 or Scenario 3. The predicted rates for RNN codons and the measured rates for YNN codons (Table 2) are used with Equation (10) to estimate the translation rate benefit of codon bias under Scenario 4.

#### Scenario 5

In contrast to the preceding scenarios that extend codon-specific translation rate measurements of YNN codons in various ways to make estimates of the effect of codon bias over all codons, Scenario 5 incorporates a theoretical prediction of the optimal translation rates for all codons based only on codon frequency and cognate tRNA abundance data. While this approach necessarily involves additional assumptions, it has the advantage of drawing on data that is more complete and less likely to be influenced by unrecognized experimental errors. Solomovici *et al*. [62] assume that selection on synonymous codon frequencies reflects intrinsic differences in rate constants for a cognate tRNA interacting with preferred and non-preferred codons, while the total tRNA abundance and amino acid composition are fixed. They demonstrate that the fastest overall translation rate is obtained when the square roots of synonymous codon frequencies are proportional to the rate constants for cognate tRNA interacting with the codons. They assume further that the rate constants for the interaction of all non-degenerate or preferred codons with their preferred cognate tRNA are identical, so the translation rate for these codons is proportional to cognate tRNA abundance. We modified the approach of reference [62] to reflect greater degeneracy in translation than assumed by the original authors ([85], also the comments earlier in this section), and applied it using the codon frequency and tRNA abundance data of Dong *et al*. [70], modified as shown in Table 2.

The predicted relative translation rates for YNN codons (i.e., the recalculated quantities *d*_*ij *_and *d*_*im, j *_of reference [62] for codons with single or multiple cognate tRNAs, respectively) are not in good agreement with observed relative rates of Curran and Yarus [45] (R^2 ^= 0.30). However, the empirical codon frequencies of Dong *et al*. [70] are correlated more closely with predicted relative rates of Scenario 5 (R^2 ^= 0.70) than with the empirical relative rates of Curran and Yarus [45] (R^2 ^= 0.31). A good correlation between the predicted translation rates and the empirical codon frequencies is expected, since the codon frequencies were used to generate the predictions. However, the poor correlation between predicted and empirical translation rates could reflect the inadequacies in any of 3 areas: 1) the assumptions of Solomovici *et al*. [62], 2) the rate measurements of Curran and Yarus [45], and/or 3) the codon and tRNA data of Dong *et al*. [70]. Alternatively, the discrepancy between predicted optimal translation rates and empirical rates may indicate that the phenotype of *E. coli *is not perfectly optimized for maximal translation rates (as suggested in reference [65]), either because of genetic drift or because of conflicting selection pressures.

Nonetheless, the disparity between the relative rates of synonymous preferred and non-preferred codons for most amino acids are greater with the predicted rates of Scenario 5 than with the observed rates. Hence, Scenario 5 will generate a higher estimate of the translation rate benefit of codon bias than would a strict application of the empirical codon-specific translation rates. (In fact, none of our scenarios are strict applications of the empirical rates; Scenarios 1–4 also deliberately extrapolate from the empirical rates in ways that will increase the estimated benefit of codon bias.) The predicted translation rates for all codons (Table 2) are used with Equation (10) to estimate the translation rate benefit of codon bias under Scenario 5.

## Authors' contributions

LD conceived of the project, collected and analyzed the data, developed the mathematical model, and drafted the manuscript. TMS helped plan the project, critiqued the work as it progressed, and edited the manuscript.

## Figures and Tables

**Table 1:** Comparisons of translational power

Comparison organism	*rrn *copy#^a^	Specific growth rate^b ^(culture)^c ^(hr^-1^)	Actual growth temp.^d ^(cor.)^e^	Compared by^f^	*E. coli *comp. growth rate^g ^(hr^-1^)	Translational power^h^	Ref.
*Sphingopyxis alaskensis*	1	0.29 (B)	30°C (1.80)	RC^i^	>1.73^j^	<17%^j^	[39]
sulfate reducing strain PT2^k^	(2)	0.40 (B)	23°C (3.34)	RNA	>1.73^j^	<17%^j^	[91]
*Streptomyces coelicolor*	6	0.54 (B)	30°C (1.80)	RNA	>1.73^j^	<21%^j^	[40]
*Halobacterium cutirubrum*	2	0.10 (B)	37°C	rRNA	0.49	22%	[37]
*Rickettsia prowazekii*	1	0.09 (B)	34°C (1.28)	RC^i^	0.37	24%	[38]
*Synechococcus *sp. 6301	(2)	0.16 (C)	39°C (0.85)	RNA	0.36	42%	[92]
*Streptomyces hygroscopicus*	(6)	0.58 – 0.90^l^ (B, C)	28°C (2.14)	RNA	0.82 – >1.73^j, l^	<42%^j ^– 110%^l^	[93]
*Megasphaera elsdenii*	-	0.20 (C)	39°C (0.85)	RNA	0.46	44%	[94]
*Bacillus cereus*	6	0.61 (C)	34°C (1.28)	RNA	1.22	51%	[95]
*Selenomonas ruminantium*	-	0.30 – 0.43^l ^(C)	39°C (0.85)	RNA	0.35 – 0.88^l^	50% – 78%^l^	[96, 97]
*Salmonella enterica*	7	1.66 (B)	37°C	rRNA	1.63	102%	[1]
*Enterobacter aerogenes*	(7)	0.94 (C)	35°C (1.18)	RNA	0.80	123% – 154%^l^	[98]
*Lactococcus lactis*	6	1.9 (B)	30°C (1.80)	RNA	0.51	391%	[99]

^a ^Number of *rrn *operons per genome were obtained from the ribosomal RNA operon copy number database [100]. Where *rrn *copy number is not available for a species, values shown in parentheses are typical for the genus or family, if such estimates are possible.

^b ^Highest growth rates (temperature corrected for 37°C, see text) at which macromolecular data were available, shown as specific growth rate = ln(2)/(*generation time*).

^c ^Culture type indicated as batch (B) or chemostat (C).

^d ^Temperature at which strains were grown for macromolecular measurements.

^e ^Correction factor applied to actual growth rate to obtain temperature corrected growth rate shown in the third column, based on data from reference [56]

^f ^Similar investment in the translational apparatus between the comparison organism and *E. coli *assessed as follows: RC, similar ribosome concentration by cell volume; RNA, similar protein:RNA ratio; rRNA, similar protein:rRNA ratio. For all comparisons, *E. coli *data were taken from reference [3] with interpolation between discrete data points as necessary.

^g ^Growth rate at which *E. coli *makes a similar investment in the translational apparatus (by the criteria in column 5) as the comparison organism at the growth rate shown in column 3.

^h ^Translational power of the comparison organism expressed as a percentage of the translational power of *E. coli*.

^i ^For consistency, the comparison of ribosome concentration between this organism and *E. coli *made in the original reference is not used. Instead, ribosome concentration as a function of growth rate in *E. coli *was calculated from the data of reference [3] assuming a cell volume of 1.1 fl at a growth rate of 1.03 hr^-1 ^and a constant ratio of cell volume to dry mass across growth rates. The comparison of translational power for this organism assumes that its protein concentration (protein mass per cell volume) is similar to *E. coli*.

^j ^Comparison organism makes a larger investment in the translational apparatus than *E. coli *growing at the fastest rate at which data are availabe. The comparison is made conservatively to data from the fastest *E. coli *growth rate.

^k ^Related to *Desulfovibrio vulgaris *by 16S rRNA gene sequence analysis.

^l ^Range of values shown corresponds to the maximum growth rates obtained for this organism in different culture conditions

**Table 2:** Codon data

Codon	AA	Codon frequency		tRNA abund.^c ^(%)	Empirical rel. trans. rates^d^	Predicted rel. translation rates			
		*E. coli*^a ^(× 10^-3^)	Low bias^b ^(× 10^-3^)			Sc. 2^e^	Sc. 3^e^	Sc. 4^e^	Sc. 5^f^

UUU	Phe	8.0	9.6	1.5	8.5	(7.6)	(6.3)	(3.2)	3.7
UUC	Phe	23.4	21.8	1.5	12.0	(12.0)	(10.8)	(10.8)	6.4
UUA	Leu	2.8	6.6	2.7	4.3	(5.1)	(5.2)	(1.9)	7.1
UUG	Leu	4.3	17.1	3.8	8.7	(6.0)	(6.5)	(6.5)	8.9
UCU	Ser	16.5	10.7	3.4	11.6	(10.3)	(9.5)	(9.5)	7.1
UCC	Ser	11.8	7.2	1.2	14.7	(9.0)	(8.0)	(7.3)	6.0
UCA	Ser	2.0	5.4	2.1	7.0	(4.6)	(3.3)	(4.6)	2.5
UCG	Ser	2.5	7.1	2.6	9.0	(5.0)	(3.7)	(0.4)	2.8
UAU	Tyr	6.8	11.3	2.7	4.3	(7.2)	(6.1)	(2.8)	7.3
UAC	Tyr	16.6	12.1	2.7	8.4	(10.4)	(9.5)	(9.5)	11.5
UGU	Cys	2.8	3.1	2.1	4.0	(5.2)	(5.4)	(1.9)	7.6
UGC	Cys	3.8	3.6	2.1	7.0	(5.8)	(6.3)	(6.3)	8.9
UGG	Trp	7.1	7.1	1.5	5.0	(7.3)	(7.3)	(7.3)	6.4
CUU	Leu	3.9	15.2	2.6	8.4	(5.8)	(3.9)	(9.8)	6.3
CUC	Leu	4.1	16.0	1.7	11.0	(5.9)	(4.1)	(12.0)	6.5
CUA	Leu	0.8	4.4	0.9	0.6	(3.6)	(0.6)	(0.6)	2.9
CUG	Leu	61.2	17.9	7.3	14.4	(18.6)	(15.7)	(15.7)	24.9
CCU	Pro	4.4	9.0	1.8	8.4	(6.1)	(4.5)	(7.3)	2.7
CCC	Pro	1.1	6.2	1.1	9.6	(3.9)	(2.3)	(0.5)	1.3
CCA	Pro	5.2	12.1	0.8	1.6	(6.5)	(4.9)	(9.0)	2.9
CCG	Pro	29.0	12.4	1.5	2.5	(13.2)	(11.6)	(11.6)	6.8
CAU	His	6.8	9.0	1.2	4.0	(7.2)	(6.3)	(2.7)	3.5
CAC	His	14.3	12.1	1.2	8.0	(9.7)	(9.1)	(9.1)	5.1
CAA	Gln	7.1	10.7	1.2	5.6	(7.3)	(5.8)	(3.4)	3.3
CAG	Gln	27.5	23.9	2.3	10.0	(12.9)	(11.4)	(11.4)	6.4
CGU	Arg	44.2	21.4	7.5	14.0	(16.0)	(13.7)	(13.7)	31.3
CGC	Arg	20.8	19.1	7.5	11.5	(11.4)	(9.4)	(10.5)	21.4
CGA	Arg	0.7	11.3	7.5	3.0	(3.5)	(1.7)	(0.6)	3.9
CGG	Arg	0.6	5.1	0.6	0.8	(3.4)	(1.6)	(0.6)	2.6

AUU	Ile	15.9	21.5	6.8		10.2	8.2	4.1	17.1
AUC	Ile	44.2	28.0	6.8		16.0	13.7	13.7	28.6
AUA	Ile	0.5	11.2	0.3		3.3	1.5	0.6	1.4
AUG	Met	21.8	21.8	1.4		11.7	10.5	10.5	5.7
ACU	Thr	20.8	15.0	3.8		11.4	9.9	8.7	7.6
ACC	Thr	26.9	18.7	1.8		12.8	11.3	11.3	8.7
ACA	Thr	2.6	10.0	2.0		5.1	3.5	0.5	2.7
ACG	Thr	4.2	10.8	2.9		6.0	4.5	7.1	3.4
AAU	Asn	5.7	16.7	2.1		6.7	5.1	3.5	3.9
AAC	Asn	29.4	18.4	2.1		13.3	11.7	11.7	8.9
AAA	Lys	55.4	39.6	3.1		17.8	15.0	15.0	12.8
AAG	Lys	17.4	33.2	1.2		10.6	8.4	4.5	2.9
AGU	Ser	2.2	6.8	1.7		4.8	3.8	2.3	3.4
AGC	Ser	9.4	7.2	1.7		8.2	7.9	7.9	7.0
AGA	Arg	0.6	5.4	1.1		3.4	4.9	0.6	3.7
AGG	Arg	0.0	4.5	1.7		2.6	1.1	0.6	0.8
GUU	Val	43.5	38.8	7.9		15.9	13.6	13.6	23.3
GUC	Val	7.7	13.2	2.0		7.5	5.7	0.6	9.8
GUA	Val	22.5	20.1	6.0		11.8	9.8	10.5	16.8
GUG	Val	15.1	16.8	6.0		9.9	8.0	8.5	13.7
GCU	Ala	39.8	38.6	7.2		15.3	13.2	13.2	19.4
GCC	Ala	11.9	15.7	1.1		9.0	7.2	0.5	10.6
GCA	Ala	25.1	30.6	6.1		12.4	10.4	10.1	15.4
GCG	Ala	24.3	16.2	6.1		12.2	10.3	8.2	15.1
GAU	Asp	19.4	22.3	4.4		11.1	9.3	3.7	14.0
GAC	Asp	34.0	31.1	4.4		14.2	12.4	12.4	18.5
GAA	Glu	58.3	39.8	8.5		18.2	15.3	15.3	35.7
GAG	Glu	17.1	35.6	3.4		10.5	8.3	4.5	7.7
GGU	Gly	45.9	23.7	9.3		16.3	13.9	13.9	21.0
GGC	Gly	34.4	23.0	7.3		14.3	12.1	10.7	18.2
GGA	Gly	1.3	17.8	1.9		4.1	2.3	0.6	3.5
GGG	Gly	2.4	19.5	3.2		4.9	3.2	8.7	4.8

^a ^Proteome codon frequencies from reference [70] for *E. coli *growing at a specific growth rate of 1.73 hr^-1^, modified slightly as described in Methods.

^b ^Low bias codon frequencies representing the degree of codon bias present in the genome of *T. pallidum*, generated as described in Methods.

^c ^Summed abundance of all tRNA species cognate to the listed codon, expressed as a percentage of total tRNA, based on tRNA abundance data of references [70] and [69] and cognate specificity of reference [85], modified slightly as described in Methods. Values for all codons sum to >100%, a result of the partially overlapping codon specificity of many tRNA species.

^d ^Empirically determined relative rates of ternary complex selection at the listed codon from reference [45], expressed relative to the rate of a uniform competing frameshift event. Rate for codons CGC and CGA modified as described in Methods. Estimates of *s*_*bias *_were made using the rates as listed or with a correction for the duration of translocation, as described in Methods.

^e ^Predicted relative translation rates based on the empirical rates of column 6 and scenarios as described in Methods. Estimates of *s*_*bias *_were made using empirical rates of column 6 for YNN codons in preference to the predicted rates shown in parentheses; predicted rates are shown for comparison only.

^f ^Predicted relative translation rates from theory of reference [62], modified slightly as described in Methods, using the codon frequency data of column 3 and the cognate tRNA abundance data of column 5.
